# Development and validation of prediction models for hypertension risks in rural Chinese populations

**DOI:** 10.7189/jogh.09.020601

**Published:** 2019-12

**Authors:** Fei Xu, Jicun Zhu, Nan Sun, Lu Wang, Chen Xie, Qixin Tang, Xiangjie Mao, Xianzhi Fu, Anna Brickell, Yibin Hao, Changqing Sun

**Affiliations:** 1 Department of Social Medicine and Health Management, College of Public Health, Zhengzhou University, Zhengzhou, Henan, PR China; 2 Department of Management Information Systems, Terry College of Business, University of Georgia, Athens, Georgia, USA; 3 College of Medicine, University of Arkansas for Medical Sciences, Little Rock, Arkansas, USA; 4 People’s Hospital of Zhengzhou, Zhengzhou, Henan, PR China

## Abstract

**Background:**

Various hypertension predictive models have been developed worldwide; however, there is no existing predictive model for hypertension among Chinese rural populations.

**Methods:**

This is a 6-year population-based prospective cohort in rural areas of China. Data was collected in 2007-2008 (baseline survey) and 2013-2014 (follow-up survey) from 8319 participants ranging in age from 35 to 74 years old. Specified gender hypertension predictive models were established based on multivariate Cox regression, Artificial Neural Network (ANN), Naive Bayes Classifier (NBC), and Classification and Regression Tree (CART) in the training set. External validation was conducted in the testing set. The estimated models were assessed by discrimination and calibration, respectively.

**Results:**

During the follow-up period, 432 men and 604 women developed hypertension in the training set. Assessment for established models in men suggested men office-based model (M1) was better than others. C-index of M1 model in the testing set was 0.771 (95% confidence Interval (CI) = 0.750, 0.791), and calibration χ^2^ = 6.3057 (*P* = 0.7090). In women, women office-based model (W1) and ANN were better than the other models assessed. The C-indexes for the W1 model and the ANN model in the testing set were 0.765 (95% CI = 0.746, 0.783) and 0.756 (95% CI = 0.737, 0.775) and the calibrations *χ^2^* were 6.7832 (*P* = 0.1478) and 4.7447 (*P* = 0.3145), respectively.

**Conclusions:**

Not all machine-learning models performed better than the traditional Cox regression models. The W1 and ANN models for women and M1 model for men have better predictive performance which could potentially be recommended for predicting hypertension risk among rural populations.

Hypertension is the leading global risk factor for premature death and disability, more than 9.4 million deaths annually, causing more than 40% of worldwide deaths from cardiovascular diseases (CVD) and chronic kidney disease [1-3]. The number of adults with hypertension has largely increased during the past four decades in low-income and middle-income countries, such as China [4,5]. Efficient primary prevention strategies targeted at individuals “at risk” are urgently needed in China to decrease the prevalence and the disease burden of hypertension [6]. Predictive models have demonstrated a cost-effective tool for identifying high-risk individuals of hypertension. One prominent hypertension prediction model, Framingham Risk Score (FRS), was developed from the Framingham Heart Study [7]. However, risk prediction models developed for one population cannot be efficiently applied to other populations or even the same country after time as societies changes [8]. Several studies have validated the FRS in different populations [9-12]. Other studies have developed their own prediction models based on FRS [13-21]. The results suggested that FRS was not a valid tool to estimate the incidence of hypertension among rural Chinese populations [12].

As a developing country, China has more than 50% of the population living in rural areas where medical and health facilities are limited. Development and validation of a hypertension risk prediction model for rural populations is an ideal and cost-effective approach for informed decision making regarding primary preventive strategies, identification, and treatment of high-risk populations. The aim of this study is to develop and validate the various hypertension risk prediction models using different algorithms and to explore whether the new algorithms could improve the performance for hypertension prediction in Chinese rural populations. Gender-specific Cox proportional hazard models, Artificial Neural Network (ANN) models, Naïve Bayes Classifier (NBC) models and Classification and Regression Tree (CART) models will be assessed and modified to find optimal models for the rural Chinese populations.

## METHODS

### Study population and samples

This survey is a 6-year population-based prospective cohort study in the rural areas of Henan Province, China (Figure S1 in **Online Supplementary Document** **s**howed the position where data was collected). Details of the study design and methods have been described and reported previously [22,23]. Briefly, the baseline survey was conducted from July to August of 2007 and of 2008. The data was collected by questionnaires, medical examinations, and fasting blood samples. Subjects were permanent residents with no major disability or severe infectious diseases. A follow-up survey was completed using the same methods from July to August of 2013 and July to October of 2014. There were 20 194 participants in the original cohort but only 17 265 participants finished the follow-up survey (including 1110 deaths). Out of 16 155 participants, 5635 participants with cancer, chronic kidney disease, hypertension, or prior history of CVD at baseline were excluded. Additionally, 672 individuals missing status on hypertension in follow-up survey were not included in analysis. Participants with a baseline age outside the age range of interest were excluded from this study (1407 persons <35 years old or >74 years old). Due to missing data, 122 participants were excluded because the calculation of risk models could not be performed. Finally, a total of 8319 participants were eligible for analysis. The participants were divided into a training set (4796 participants) and a testing set (3523 participants) according to their different living areas. This study was approved by the Medical Ethics Committee of Zhengzhou University. All participants signed an informed-consent form.

### Data collection and laboratory measurements

Data was collected through standardized methods under stringent quality control conducted by specially trained physicians and public health workers. The information regarding demographic characters such as family and individual disease history, diet, and lifestyle were obtained by a standardized questionnaire. Anthropometric data was also included: height, weight, waist circumference (WC), hip circumference, systolic blood pressure (SBP), and diastolic blood pressure (DBP). WC and hip circumference were both measured twice. Blood pressure was measured three times by HEM-770A sphygmomanometer in the sitting position according to the American Heart Association’s standardized protocol [24], and the mean of the 3 blood pressure measurements was calculated and used for analysis. Blood specimens were collected for measurement of lipid profiles and fasting plasma glucose levels following overnight fasting. Hypertension was defined as SBP≥140 mm Hg and/or DBP≥90 mm Hg, and/or a diagnosis of hypertension by a physician and currently receiving anti-hypertension treatment according to 20l0 Chinese guidelines for the management of hypertension [25]. Type 2 Diabetes Mellitus (T2DM) status was defined as having a fasting plasma glucose (FPG)≥7.0 mmol/L, and/or a diagnosis of diabetes by a physician [26].

### Prediction models selection and development

Exploration of novel approaches for disease prevention is ongoing. In recent years, machine learning approaches have been introduced in disease prediction especially in accessing the accuracy of the CVD risk prediction. Various studies indicated that some machine learning models have better accuracy, more advantages in computing power, and the ability of handle complex variables compared to the traditional statistical models [27-30]. The Cox regression model and three machine learning models (ANN model, NBC model and CART model) were chosen to develop the optimal hypertension risk model for rural Chinese populations.

Univariate analysis was performed to evaluate candidate predictors of hypertension and prediction models were established in the training set. Multivariate Cox regression was used to established gender-specific laboratory-based and office-based hypertension prediction models. Gender-specific ANN models, NBC models, and CART models were also placed in the training set. In addition, 10-fold cross-validation technique was conducted for all established models in the process of model development to make the models more stable and accurate [31-33]. Then “external validation” of all established models was conducted for the testing set.

### Statistical analysis

The whole process of statistical analysis was performed with the R software (version 3.4.1, https://www.R-project.org). Continuous variables were described by mean ± standard deviation (if normally distributed) or median (inter-quartile range) (if not normally distributed), while categorical data was reported as quantity and percentages. Validity and predictive accuracy of the hypertension risk models was assessed based on their discrimination and calibration. A 2-tailed *P*-value <0.05 was considered significant.

The C-index was calculated to evaluate the discriminative power of risk models. A C-index is the area under the receiver operating characteristic (ROC) curve (AUC). Calibration was assessed by modified Nam-D’Agostino tests [34,35] to determine whether the observed hypertension events differed significantly from the expected. Internal validation of the discrimination and calibration performance was evaluated by 10-fold cross-validation technique in the training set [31-33], and the external validation was conducted in the testing set.

## RESULTS

### Baseline characteristics

The demographic and clinical characteristics of the participants at baseline are presented in **Table 1****,** and Tables S1 and S2 in **Online Supplementary Document****.** In the training set, there were 4796 participants (1853 men and 2943 women). The 6-year follow-up survey revealed 1036 individuals (432 men and 604 women) developed hypertension with a duration of 27799.5 person-years in the training set. Incidence densities of hypertension were 40.3964 and 35.3113 per 1000 person-years for men and women, respectively. In the training set, men had older age, higher SBP, DBP smoking rates, larger WC, and pulse pressure than women. However, total cholesterol (TC), high density lipoprotein cholesterol (HDL-c), FPG, T2DM incidence and body mass index (BMI) were more prevalent in women than in men. Univariate analysis of the Cox proportional hazards regression model in the training set was presented in Table S3 in **Online Supplementary Document** (for men) and Table S4 in **Online Supplementary Document** (for women).

**Table 1:** Baseline demographic characteristics and biochemical indexes of the training set

Variables*	Men (n = 1853)	Women (n = 2943)	*P*-value
**Age, years**	52 (44-59)	48 (41-56)	<0.0001†
**Educational level (n, %):**			<0.0001‡
Illiteracy	93 (5.02)	516 (17.53)	
Primary school	511 (27.58)	1072 (36.43)	
Junior high	951 (51.32)	1162 (39.48)	
High school and above	298 (16.08)	193 (6.56)	
**Marital status (n, %):**			0.0463‡
Married/cohabitation	1721 (93.08)	2782 (94.53)	
Others	128 (6.92)	161 (5.47)	
**Income§, CNY (n, %):**			0.0014‡
<1000	1675 (90.59)	2747 (93.44)	
1000 ~	131 (7.08)	142 (4.83)	
≥3000	43 (2.33)	51 (1.73)	
Hypertension paternal history (n, %)	509 (27.47)	882 (29.97)	0.0679‡
High fat diet (n, %)	144 (7.77)	43 (1.46)	<0.0001‡
Fruit and vegetable intake (n, %)	860 (46.41)	1137 (38.63)	<0.0001‡
General obesity (n, %)	160 (8.63)	399 (13.56)	<0.0001‡
Central obesity (n, %)	420 (22.67)	1564 (53.14)	<0.0001‡
Current smoking (n, %)	1143 (61.68)	9 (0.31)	<0.0001‡
Drink (n, %)	585 (31.57)	16 (0.54)	<0.0001‡
T2DM (n, %)	98 (5.29)	234 (7.95)	0.0005‡
Heart rate, bpm	70 (64-78)	75 (69-82)	<0.0001†
SBP, mm Hg	118 (110-126)	115 (107-124)	<0.0001†
TC, mmol/L	4.26 (3.76-4.85)	4.42 (3.88-5.05)	<0.0001†
TG, mmol/L	1.20 (0.90-1.80)	1.30 (0.90-1.80)	0.2300†
HDL-c, mmol/L	1.09 (0.94-1.27)	1.19 (1.02-1.37)	<0.0001†
LDL-c, mmol/L	2.50 (2.10-3.00)	2.50 (2.10-3.00)	0.0860†
FPG, mmol/L	5.30 (4.94-5.71)	5.33 (4.99-5.75)	0.0059†
DBP, mm Hg	74.67 (68.67-80.00)	73.67 (68.67-79.00)	0.0250†
Pulse pressure, mm Hg	43.67 (38.67-49.00)	41.00 (35.67-47.00)	<0.0001†
BMI, kg/m^2^	23.32 (21.19-25.49)	24.17 (21.94-26.53)	<0.0001†
WC, cm	81.40 (75.25-89.10)	80.75 (74.00-87.23)	<0.0001†

T2DM – type 2 diabetes mellitus, SBP – systolic blood pressure, TC – total cholesterol, TG – triglyceride, HDL-c – high-density lipoprotein cholesterol, LDL-c – low-density lipoprotein cholesterol, FPG – fasting plasma glucose, DBP – diastolic blood pressure, BMI – body mass index, WC – waist circumference, CNY – Chinese Yuan, bpm – beats per minute

*Data are numbers (percent) for categorical variables and median (interquartile range) median (interquartile range) for continuous variables.

†Wilcoxon rank sum test.

**‡**χ^2^ test.

§Average monthly income.

### Development of predictive models

#### Office-based model

Gender specified office-based Cox regression models were established in the training set. Based on the results of the univariate analysis age, SBP, DBP, pulse pressure, WC, BMI, current smoking status, hypertension parental history, educational level, and available interaction between age with other risk factors (SBP, DBP, pulse pressure, WC, BMI, current smoking status, and hypertension parental history) were considered for the men office-based model (M1). Subsequently, age, SBP, DBP, hypertension parental history, WC, interaction item of age with WC, and interaction item of age with DBP were included in M1 model (**Table 2****)**. Cox regression for women office-based model (W1) was established in the same way. Lastly, age, SBP, DBP, WC, fruit and vegetable intake, hypertension parental history, interaction item of age with WC and interaction of age with DBP were included in W1 model (**Table 2****)**.

**Table 2:** Cox regression models for hypertension in men and women

Variables	β	HR (95% CI)	*P*-value
**M1 model:**			
Age, years	0.2650	1.3035 (1.1597, 1.4651)	<0.0001
SBP, mmHg	0.0554	1.0570 (1.0429, 1.0712)	<0.0001
DBP, mmHg	0.1300	1.1388 (1.0532, 1.2314)	0.0011
WC, cm	0.0626	1.0646 (1.0095, 1.1228)	0.0209
hypertension paternal history (Yes vs No)	0.3441	1.4107 (1.1463, 1.7361)	0.0012
Age × WC*	-0.0011	0.9989 (0.9980, 0.9999)	0.0264
Age × DBP†	-0.0019	0.9981 (0.9967, 0.9995)	0.0067
**W1 model:**			
Age, years	0.3430	1.4092 (1.2722, 1.5608)	<0.0001
SBP, mmHg	0.0525	1.0539 (1.0425, 1.0654)	<0.0001
DBP, mmHg	0.1956	1.2161 (1.1356, 1.3023)	<0.0001
WC, cm	0.0807	1.0840 (1.0347, 1.1357)	0.0007
Higher vegetables and fruit intake (Yes vs No)	-0.1345	0.8742 (0.7375, 1.0363)	0.1212
hypertension paternal history (Yes vs No)	0.2189	1.2447 (1.0417, 1.4872)	0.0159
Age × WC*	-0.0013	0.9987 (0.9978, 0.9995)	0.0020
Age × DBP†	-0.0026	0.9974 (0.9962, 0.9986)	<0.0001
**W2 model:**			
Age, years	0.3413	1.4068 (1.2703, 1.5579)	<0.0001
SBP, mmHg	0.0525	1.0539 (1.0425, 1.0654)	<0.0001
DBP, mmHg	0.1943	1.2144 (1.1340, 1.3005)	<0.0001
WC, cm	0.0799	1.0832 (1.0338, 1.1349)	0.0008
Higher vegetables and fruit intake (Yes vs No)	-0.1356	0.8732 (0.7366, 1.0351)	0.1181
hypertension paternal history (Yes vs No)	0.2094	1.2330 (1.0318, 1.4734)	0.0212
Age × WC*	-0.0014	0.9986 (0.9977, 0.9995)	0.0018
Age × DBP†	-0.0026	0.9974 (0.9962, 0.9986)	<0.0001
HDL-c, mmol/L	-0.2807	0.7752 (0.5822, 0.9796)	0.0344

SBP – systolic blood pressure, DBP – diastolic blood pressure, HDL-c – high-density lipoprotein cholesterol, WC – waist circumference, HR – hazard ratio, M1 – men office-based model, W1 – women office-based model, W2 – women laboratory-based model, CI – confidence interval

*Interaction item of age with WC.

†Interaction item of age with DBP.

#### Laboratory-based model

Gender specific laboratory-based models were conducted based on office-based models. Biochemical factors and available interaction terms of age with biochemical factors were added as covariates. The findings indicated that no biochemical factors were included in the men laboratory-based model. Thus, the laboratory-based model for men was the same as the M1 model. In addition, women laboratory-based model (W2) added HDL-c compared to W1 model (**Table 2****)**. The Cox regression model indicated good internal consistency (accessed by 10-fold cross-validation) in the training set (Table S5 in **Online Supplementary Document****)**.

#### Machine learning models

For both genders, the ANN models included age, SBP, DBP, parental hypertension history, and BMI as predictors in the input layer. A 10-fold cross-validation indicated that three nodes in the hidden layer for men and nine nodes in the hidden layer for women made the models have a decreased root-mean-square-error (RMSE) as shown in Figure S2 and Figure S3 in **Online Supplementary Document****.** The NBC models included age, SBP, DBP, parental hypertension history, and BMI as predictors for both genders. Only SBP was included for both genders in the CART model (Figure S4 in **Online Supplementary Document****)**. According to the results of the 10-fold cross-validation, the complexity parameter was set as 0.012 to make the RMSE lower for (Figure S5 in **Online Supplementary Document****)** both genders than the RMSA for the CART model.

### Model performance

#### Model performance in training set

**Figure 1:**
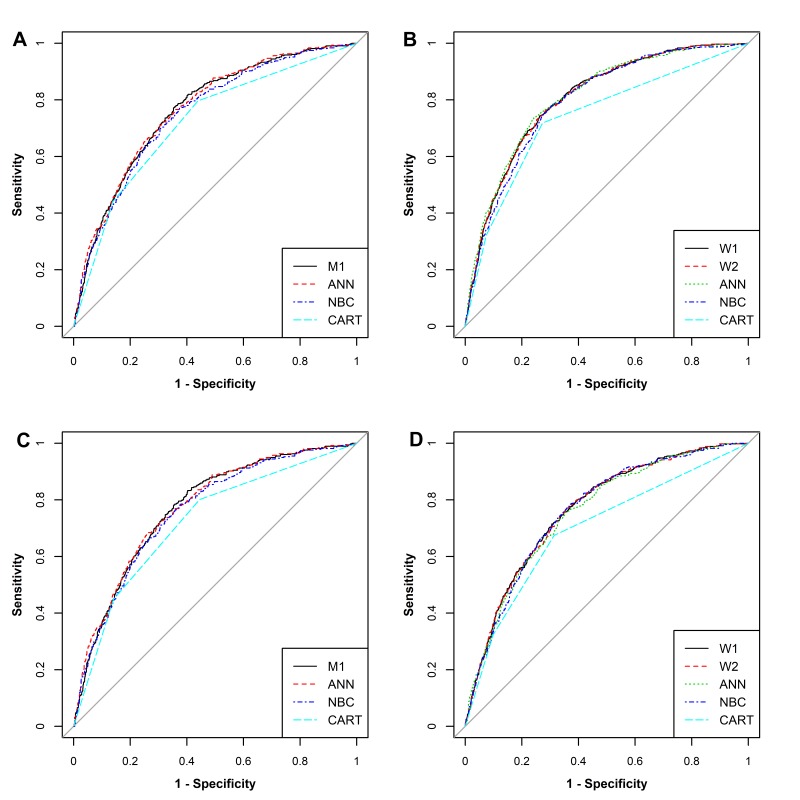
ROC curves of different models for prediction of hypertension incidence in the training and testing set. **Panel A** shows ROC curves of different models for prediction of hypertension incidence for men in training set. **Panel B** shows ROC curves of different models for prediction of hypertension incidence for women in training set. **Panel C** shows ROC curves of different models for prediction of hypertension incidence for men in testing set. **Panel D** shows ROC curves of different models for prediction of hypertension incidence for women in testing set.

ROC curves of different hypertension risk prediction models for the training set are shown in **Figure 1** **(**Panel A for men, and Panel B for women**)**. AUCs of nine hypertension predictive models (4 for men and 5 for women) showed moderate discrimination (**Table 3**, Table S6 and Table S7 in **Online Supplementary Document**). In men, the AUCs ranged from 0.720 (95% Confidence Interval, 95%CI = 0.699, 0.741) in the CART model to 0.767 (95%CI = 0.747, 0.786) in the ANN model. In women, the AUCs arranged from 0.740 (95%CI = 0.724, 0.756) in the CART model to 0.809 (95%CI = 0.795, 0.823) in the ANN model.

**Table 3:** Discriminative ability and calibration of the different 6-year hypertension incident risk models for both genders in training and testing set, respectively

Models	Cut-off	AUC (95% CI)	Calibration χ^2^	*P*-value
**Training set:**				
**Men**				
M1	0.1926	0.765 (0.745, 0.784)	4.91334	0.84180
ANN	0.2305	0.767 (0.747, 0.786)	24.54347	0.00352
NBC	0.2205	0.751 (0.730, 0.770)	105.88180	<0.00001
CART	0.0994	0.720 (0.699, 0.741)	4.56824	0.10186
**Women**				
W1	0.1920	0.806 (0.791, 0.820)	4.72712	0.31645
W2	0.1922	0.806 (0.791, 0.820)	1.18206	0.88104
ANN	0.2512	0.809 (0.795, 0.823)	5.44370	0.24472
NBC	0.2588	0.796 (0.780, 0.810)	193.18980	<0.00001
CART	0.0909	0.740 (0.724, 0.756)	17.95192	0.00012
**Testing set:**				
**Men**				
M1	0.1745	0.771 (0.750, 0.791)	6.30570	0.70898
ANN	0.2799	0.773 (0.752, 0.793)	29.27430	0.00058
NBC	0.2205	0.760 (0.738, 0.781)	82.26996	<0.00001
CART	0.0994	0.722 (0.699, 0.743)	5.249259	0.07247
**Women**				
W1	0.1798	0.765 (0.746, 0.783)	6.78323	0.14780
W2	0.1446	0.764 (0.746, 0.783)	7.40462	0.11599
ANN	0.2022	0.756 (0.737, 0.775)	4.74466	0.31451
NBC	0.1860	0.761 (0.742, 0.779)	189.75400	<0.00001
CART	0.0909	0.698 (0.677, 0.717)	19.73303	0.00005

AUC – area under the receiver operating characteristic curve, CI – confidence interval, M1 – men office-based model, ANN – Artificial Neural Network, NBC – Naive Bayes Classifier, CART – Classification and Regression Tree, W1 – women office-based model, W2 – women laboratory-based model

According to the modified Nam-D’Agostino test, the M1 model (χ^2^ = 4.9133, *P* = 0.8418) and CART (χ^2^ = 4.5682, *P* = 0.1019) model for men showed good agreement between the predicted and observed hypertension events in the training set. The ANN model and NBC model overestimated the hypertension risk (Figure S6 in **Online Supplementary Document**, **Table 3** and Table S8 in **Online Supplementary Document**). For women, the W1 model (χ^2^ = 4.7272, *P* = 0.3165), W2 model (χ^2^ = 1.1821, *P* = 0.8810), and ANN model (χ^2^ = 5.4478, *P* = 0.2447) all showed good agreement between the predicted and observed hypertension events in the training set whereas the NBC model and CART model overestimated the risk. (Figure S7 in **Online Supplementary Document**, **Table 3** and Table S9 in **Online Supplementary Document**).

#### 3.3.2 Model performance in testing set

ROC curves of different models for testing set were shown in **Figure 1** **(**Panel C for men and Panel D for women**)**. AUCs of nine models showed moderately good discrimination (**Table 3**, Table S10 and Table S11 in **Online Supplementary Document****)**. In men, the AUCs ranged from 0.722 (95%CI = 0.699, 0.743) in the CART model to 0.773 (95%CI = 0.752, 0.793) in the ANN model. In women, the AUCs arranged from 0.698 (95%CI = 0.677, 0.717) in the CART model to 0.765 (95%CI = 0.746, 0.783) in the ANN model.

According to the modified Nam-D’Agostino test, M1 (χ^2^ = 6.3057, *P* = 0.7090) the model for men showed good agreement between the predicted and observed hypertension events in the testing set. The ANN model, NBC model, and CART model for men overestimated the hypertension risk in testing set (Figure S8 in **Online Supplementary Document****,** **Table 3** and Table S12 in **Online Supplementary Document****)**. Notably, the W1 model (χ^2^ = 6.7832, *P* = 0.1478), W2 model (χ^2^ = 7.4046, *P* = 0.1160) and ANN model (χ^2^ = 4.7747, *P* = 0.3145) for women showed good agreement between the predicted and observed hypertension events in the testing set. The NBC model and CART model for women overestimated the risk in testing set (Figure S9 in **Online Supplementary Document****,** **Table 3** and Table S13 in **Online Supplementary Document****)**.

## DISCUSSION

This study evaluated the ability of nine hypertension risk models in rural Chinese populations. All nine models performed well in discrimination. M1 model was well-calibrated for men, while the W1 model, W2 model, and ANN model showed an appropriate calibration for women.

Risk predictive models are essential and cost-effective for prevention of hypertension especially in the rural regions of China where resources are limited. Readily available, unbiased predictors were considered for different models in this study. Investigations demonstrated the genetic risk score (GRS) which represented genetic factors were independently associated with elevated blood pressure and hypertension incidence [36,37]. Unfortunately, measurements of GRS were absent in this study and therefore genetic factor relevance was compared to positive family history of hypertension [36]. Thus, parental hypertension history was considered the available genetic factors for individuals in the predictive models.

Existing hypertension models around the world were not developed from rural populations [7,13-19,21], except one Indian study [20]. This study developed different prediction models for hypertension in rural Chinese populations based on a prospective cohort. Individuals included in this study ranged in age from 35 years old to 74 years old. There was a higher incidence of hypertension with increased age compared to the younger participants. Additionally, gender-specific models were established as the different levels of prevalence for hypertension risk factors between men and women. Smoking was not included as a predictor in the present study compared to the FRS model [7] because the female smoking rate was significantly lower (0.31% in training set and 0.24% in testing set) than that of men (61.68% in training set and 59.09% in testing set) in this population. Previous investigations have demonstrated that machine learning approaches have a suitable performance in cardiovascular diseases and mortality prediction [28-30], similar to the results of this study. The ANN model and NBC model in both genders had a good discrimination (C-index were more than 0.77) but only the ANN model for women had an accepted calibration. This may primarily be due to the machine learning algorithm models (ANN, NBC and CART) inability to fully utilize the time variable in the cohort study to deal with censored, time-to-event data. Machine learning approaches need to be further explored and improved [38].

The FRS model for hypertension performed poorly in rural Chinese regions [12]. The C-index for two- and four-year incidence of hypertension was respectively 0.537 (95% CI = 0.524, 0.550) and 0.610 (95% CI = 0.602, 0.618) which was lower than the findings in this study. Calibration of the FRS model was inadequate in the rural Chinese population leading to believe the FRS model cannot be applied efficiently. These findings may be due to the fact the FRS model was developed from individuals of Caucasian ethnicity. Validation and recalibration should be conducted before this risk assessment tool can be applied in other populations [23]. Existing models mostly included hypertension predictors based on the FRS model [13-20], which was easily available. A prospective northern urban Han Chinese cohort study indicated suitable discrimination in the prediction model for risk of incident hypertension, in which the AUC was 0.760 (95% CI = 0.751, 0.770) for men and 0.749 (95% CI = 0.737, 0.761) for women [21]. However, the predictors in that study such as gamma-glutamyl transferase, TC, and neutrophil granulocytes were not easily obtainable for rural individuals. Therefore, the performance of this model and the absolute risk of hypertension incidence could not be assessed as it did not provide the calibration [21].

Previous investigations of hypertension risk models were utilized for internal validation by dividing participants proportionately into two groups: training set and testing set [13,14]. However, the results for internal validation were not compelling proof of evidence for the external application. Similar to previous investigations, internal validations were conducted using a 10-fold cross-validation method for all established models in this study. This could improve the stability and avoid the phenomenon of over-fitting in these models [31-33]. External validations were also performed for all established models in the testing set to assess the generalized ability and application of the models.

In this study, the M1 model for men, ANN model and NBC model had fitting discriminations, but only the M1 model calibrated well for the testing set. The W1 model, W2 model, ANN model, and NBC model discriminated well for women. Only the W1 model, W2 model, and ANN model had appropriate calibrations in the testing set. The W1 model and W2 model were both assessed, and no significant difference was observed between them. These findings indicate that the W1 model without laboratory parameters can be used broadly including the low-income regions. Thus, in rural Chinese populations the M1 model could predict the risk of incidence of hypertension accurately and should be recommended to assess risk in men. Likewise, the W1 model and ANN model could accurately predict the hypertension risk for women.

### Strengths and limitations

Based on the prospective cohort, this study collected data from a relatively large-scale population in a rural Chinese region and developed different hypertension risk models. There were 1855 individuals that developed hypertension during follow-up survey, therefore, number of events met a ratio of at least 10 events per variable, which could avoid the overfitting of the Cox regression model [39,40]. Using machine learning approaches, internal and external validation were conducted and assessed in this study. Furthermore, calibrations were performed with modified Nam-D’Agostino tests which could deal with censored and surviving data. Although it is the first time to assess different hypertension risk prediction in rural Chinese populations, some limitations need to be addressed. First, information bias such as recall bias and loss to follow-up bias could exist in this study which cannot be avoided in observational studies. Second, hypertension has been associated with various factors and this study only included the most important available factors as predictors for hypertension risk. Third, the study was conducted in a single rural area and the results will possibly need to be validated on a larger population in a multicenter study. Although this study has several limitations, exceptional efforts were made to modify and critique the models. This study represents the actuality of hypertension risk in the rural Chinese population and the results should be considered relatively accurate and reliable.

## CONCLUSION

This study highlighted that not all of the modern machine-learning models performed better than Cox regression models. The W1 and ANN model in women and M1 model in men have a more efficient predictive capability and could be recommended for predicting hypertension risk among rural populations.

## Additional material

Online Supplementary Document
