# Supplementary material for: Development and validation of prediction models for hypertension risks in rural Chinese populations
Source: J Glob Health. 2019 Nov 24;9(2):020601. doi: 10.7189/jogh.09.020601 (PMC6875679; doi:10.7189/jogh.09.020601)
Supplement: Online Supplementary Document [file jogh-09-020601-s001.pdf]

Online Supplementary Document

Title:

Development and Validation of Prediction Models for Hypertension Risks in Rural Chinese Populations

Running Head

Hypertension Risk Model for rural China

Authors

Fei Xu [1], Jicun Zhu [1], Nan Sun [2], Lu Wang [1], Chen Xie [1], Qixin Tang [1], Xiangjie Mao [1], Xianzhi Fu [1], Anna Brickell [3], Yibin Hao [4] and Changqing Sun [1*]

Authors affiliations

[1] Department of Social Medicine and Health Management, College of Public Health, Zhengzhou University, Zhengzhou, Henan, *PR* China.

[2] Department of Management Information Systems, Terry College of Business, University of Georgia, Athens, Georgia, United State.

[3] College of Medicine, University of Arkansas for Medical Sciences, Little Rock, Arkansas, United State.

[4] People's Hospital of Zhengzhou, Zhengzhou, Henan, PR China.

*** Corresponding author**

Prof. Chang-qing Sun

Department of Social Medicine and Health Management,

College of Public Health, Zhengzhou University,

100 Kexue Avenue, Zhengzhou, 450001, Henan, *PR* China.

E-mail: zzugwsy@163.com

Online Supplementary Document

Supplementary Table S1 Baseline demographic characteristics and biochemical indexes of study participants.

Supplementary Table S2 Baseline demographic characteristics and biochemical indexes of testing set.

Supplementary Table S3 Univariate Cox proportional hazards regression analysis for men in training set.

Supplementary Table S4 Univariate Cox proportional hazards regression analysis for women in training set.

Supplementary Table S5 Cross-validation of Cox regression models (M1, W1 and W2 model) for internal consistency of hypertension incident risk in training set.

Supplementary Table S6 Discriminative ability, sensitivity and specificity of the different 6-year hypertension incident risk models for men in training set.

Supplementary Table S7 Discriminative ability, sensitivity and specificity of the different 6-year hypertension incident risk models for women in training set.

Supplementary Table S8 Calibration of Kaplan-Meier observed and predicted 6-year hypertension incident for men in training set.

Supplementary Table S9 Calibration of Kaplan-Meier observed and predicted 6-year hypertension incident for women in training set.

Supplementary Table S10 Discriminative ability, sensitivity and specificity of the different 6-year hypertension incident risk models for men in testing set.

Supplementary Table S11 Discriminative ability, sensitivity and specificity of the different 6-year hypertension incident risk models for women in testing set.

Supplementary Table S12 Calibration of Kaplan-Meier observed and predicted 6-year hypertension incident for men in testing set.

Supplementary Table S13 Calibration of Kaplan-Meier observed and predicted 6-year hypertension incident for women in testing set.

Supplementary Figure S1 Sketch map of data collection site.

Supplementary Figure S2 Nodes in hidden layer for men (A) and women (B) in ANN models according to 10×10 cross-validation.

Supplementary Figure S3 Network chat of ANN models. There are three nodes in hidden layer for men (A) and nine nodes in hidden layer for women (B), and line

thickness and color gradation represent the weight of the predictors. FamHTN1 means hypertension parental history and HTN means hypertension.

Supplementary Figure S4 Tree graph of CART models for men (A) and women (B).

Supplementary Figure S5 Complexity parameter for men (A) and women (B) in CART models according to 10-fold cross-validation.

Supplementary Figure S6 Kaplan-Meier observed and predicted hypertension incident for different hypertension models in training set men: M1 model (A), ANN model (B), CART model (C) and NBC model (D), by groups of predicted probabilities.

Supplementary Figure S7 Kaplan-Meier observed and predicted hypertension incident for different hypertension models in training set women: W1 model (A), W2 model (B), ANN model (C), CART model (D) and NBC model (E), by groups of predicted probabilities.

Supplementary Figure S8 Kaplan-Meier observed and predicted hypertension incident for different hypertension models in testing set men: M1 model (A), ANN model (B), CART model (C) and NBC model (D), by groups of predicted probabilities.

Supplementary Figure S9 Kaplan-Meier observed and predicted hypertension incident for different hypertension models in testing set women: W1 model (A), W2 model (B), ANN model (C), CART model (D) and NBC model (E), by groups of predicted probabilities.

Supplementary Table S1 Baseline demographic characteristics and biochemical indexes of study participants.

Variables	Training set (n = 4796)	Testing set (n = 3523)	*P*
Age, years	50 (42-57)	50 (43-58)	0.3819§
Gender, men (n, %)	1853 (38.64)	1447 (41.07)	0.0263‖
Educational level (n, %)			0.5687‖
Illiteracy	609 (12.70)	438 (12.43)	
Primary school	1583 (33.01)	1208 (34.29)	
Junior high	2113 (44.06)	1539 (43.68)	
High school and above	491 (10.24)	338 (9.59)	
Marital status (n, %)			0.0169‖
Married/cohabitation	4503 (93.97)	3354 (95.20)	
Others	289 (6.03)	169 (4.80)	
Income *, CNY † (n, %)			<0.0001‖
< 1000	4422 (92.34)	3373 (95.91)	
1000 ~	273 (5.70)	130 (3.70)	
≥ 3000	94 (1.96)	14 (0.40)	
hypertension Paternal history (n, %)	1391 (29.00)	1034 (29.35)	0.7495‖
High fat diet (n, %)	187 (3.90)	171 (4.85)	0.0389‖
Fruit and vegetable intake (n, %)	1997 (41.64)	1381 (39.20)	0.0027‖
General obesity (n, %)	559 (11.66)	343 (9.74)	0.0060‖
Central obesity (n, %)	1984 (41.37)	1335 (37.90)	0.0015‖
Current smoking (n, %)	1152 (24.02)	860 (24.41)	0.6998‖
Drink (n, %)	601 (12.53)	398 (11.30)	0.0936‖
T2DM (n, %)	332 (6.92)	218 (6.19)	0.1979‖
Heart rate, bpm ‡	74 (67-84)	74 (67-81)	0.0114§
SBP, mm Hg	116 (108-125)	117 (109-125)	0.1470§
TC, mmol/L	4.35 (3.83-4.98)	4.44 ± 0.95	0.5597§
TG, mmol/L	1.30 (0.90-1.80)	1.40 (1.00-2.00)	<0.0001§
HDL-c, mmol/L	1.15 (0.99-1.33)	1.13 (0.99-1.31)	0.0588§
LDL-c, mmol/L	2.50 (2.10-3.00)	2.50 (2.03-3.00)	0.0091§
FPG, mmol/L	5.32 (4.97-5.74)	5.33 (5.00-5.73)	0.0194§
DBP, mm Hg	74.00 (68.67-79.33)	74.33 (69.33-79.67)	0.0056§
Pulse pressure, mm Hg	42.00 (37.00-48.00)	42.00 (36.67-47.67)	0.9841§
BMI, kg/m^2	23.83 (21.65-26.18)	23.43 (21.21-25.26)	<0.0001§
WC, cm	81.00 (74.40-88.00)	80.25 (73.75-87.00)	0.0010§

Data is quantity (percentage) for categorical variables and median (interquartile range) for continuous variables.

* Average monthly income, † CNY: Chinese Yuan, ‡ bpm: beats per minute. § Wilcoxon rank sum test. ‖ Chi-square test.

Abbreviations: T2DM, type 2 diabetes mellitus; SBP, systolic blood pressure; TC, total cholesterol; TG, triglyceride; HDL-c, high-density lipoprotein cholesterol; LDL-c, low-density lipoprotein cholesterol; FPG, fasting plasma glucose; DBP, diastolic blood pressure; BMI, body mass index; WC, waist circumference.

Supplementary Table S2 Baseline demographic characteristics and biochemical indexes of testing set.

Variables	Men (n = 1447)	Women (n = 2076)	*P*
Age, years	52 (44-60)	48 (42-56)	<0.0001[§]
Educational level (n, %)			0.5687[‖]
Illiteracy	57 (3.94)	381 (15.35)	
Primary school	424 (29.30)	784 (37.76)	
Junior high	767 (53.01)	772 (37.19)	
High school and above	199 (13.75)	139 (6.70)	
Marital status (n, %)			0.0169[‖]
Married/cohabitation	1372 (94.82)	1982 (95.47)	
Others	75 (5.18)	94 (4.53)	
Income[*], CNY[†] (n, %)			<0.0001[‖]
< 1000	1386 (95.78)	1987 (95.99)	
1000 ~	57 (3.94)	73 (3.53)	
≥ 3000	4 (0.28)	10 (0.48)	
hypertension paternal history (n, %)	117 (8.09)	54 (2.60)	0.7495[‖]
High fat diet (n, %)	149 (9.00)	72 (2.99)	0.0389[‖]
Fruit and vegetable intake (n, %)	588 (40.64)	793 (38.20)	0.0027[‖]
General obesity (n, %)	87 (6.01)	256 (12.33)	0.0060[‖]
Central obesity (n, %)	304 (21.01)	1031 (49.66)	0.0015[‖]
Current smoking (n, %)	855 (59.09)	5 (0.24)	0.6998[‖]
Drink (n, %)	383 (26.47)	15 (0.72)	0.0936[‖]
T2DM (n, %)	88 (6.08)	130 (6.26)	0.1979[‖]
Heart rate, bpm[‡]	71 (65-78)	76 (70-83)	<0.0001[§]
SBP, mm Hg	117 (110-126)	116 (108-125)	0.0031[§]
TC, mmol/L	4.29 (3.77-4.89)	4.39 (3.83-5.00)	0.0008[§]
TG, mmol/L	1.40 (1.00-2.00)	1.40 (1.00-2.00)	0.9366[§]
HDL-c, mmol/L	1.08 (0.95-1.24)	1.17 (1.02-1.45)	<0.0001[§]
LDL-c mmol/L	2.50 (2.00-3.00)	2.50 (2.10-3.00)	0.6503[§]
FPG, mmol/L	5.29 (4.99-5.70)	5.35 (5.02-5.76)	0.0193[§]
DBP, mm Hg	74.00 (69.00-79.67)	74.67 (69.33-79.67)	0.0754[§]
Pulse pressure, mm Hg	43.00 (38.33-48.33)	41.33 (35.92-47.42)	<0.0001[§]
BMI, kg/m^2	22.85 (20.80-24.95)	23.98 (21.59-26.21)	<0.0001[§]
WC, cm	81.20 (74.25-88.10)	79.90 (73.35-86.50)	<0.0001[§]

Data is quantity (percentage) for categorical variables and median (interquartile range) for continuous variables.

[*] Average monthly income, [†] CNY: Chinese Yuan, [‡] bpm: beats per minute. [§] Wilcoxon rank sum test. [‖] Chi-square test.

Abbreviations: T2DM, type 2 diabetes mellitus; SBP, systolic blood pressure; TC, total cholesterol; TG, triglyceride; HDL-c, high-density lipoprotein cholesterol; LDL-c, low-density lipoprotein cholesterol; FPG, fasting plasma glucose; DBP, diastolic blood pressure; BMI, body mass index; WC, waist circumference.

Supplementary Table S3 Univariate Cox proportional hazards regression analysis for men in training set.

Variables	*β*	*HR* (95%*CI*)	*P*
Age, years	0.0304	1.0310 (1.0210, 1.0410)	<0.0001
SBP, mmHg	0.0733	1.0760 (1.0650, 1.0870)	<0.0001
DBP, mmHg	0.0791	1.0820 (1.0680, 1.0970)	<0.0001
Pulse pressure, mmHg	0.0580	1.060 (1.0470, 1.0730)	<0.0001
WC, cm	0.0201	1.020 (1.01100, 1.0300)	<0.0001
BMI, kg/m^2	0.0714	1.0740 (1.0430, 1.1060)	<0.0001
Heart rate, bpm	0.0018	1.0020 (0.9924, 1.0110)	0.7110
T2DM (Yes vs No)	0.3107	1.3640 (0.9467, 1.9660)	0.0957
Current smoking (Yes vs No)	-0.2084	0.8119 (0.6710, 0.9824)	0.0321
Drink (Yes vs No)	-0.0464	0.9546 (0.7768, 1.1730)	0.6590
Higher vegetables and fruit intake (Yes vs No)	0.0260	1.0260 (0.9743, 1.2400)	0.7870
High fat diet (Yes vs No)	0.2908	1.3375 (0.9729, 1.8390)	0.0734
hypertension paternal history (Yes vs No)	0.2686	1.3081 (1.0700, 1.5990)	0.0088
General obesity (Yes vs No)	0.1843	1.2024 (0.8746, 1.6530)	0.2560
Central obesity (Yes vs No)	0.4529	1.5728 (1.2800, 1.9330)	<0.0001
Weight categories			
Low weight	-0.3135	0.7309 (0.3607, 1.4810)	0.3843
Normal weight		1.00	
Over weight	0.4039	1.4976 (1.2246, 1.8310)	<0.0001
Obesity	0.3379	1.4020 (1.1006, 1.9540)	0.0459
Income [*], CNY [†] (n, %)			
< 1000		1.00	
1000 ~	-0.0082	0.9918 (0.6841, 1.4380)	0.9650
≥ 3000	0.0027	1.0027 (0.5352, 1.8780)	0.9930
Educational level (n, %)			
Illiteracy		1.00	
Primary school	-0.1474	0.8630 (0.5751, 1.2949)	0.4766
Junior high	-0.4610	0.6307 (0.4244, 0.9371)	0.0225
High school and above	-0.3465	0.7072 (0.4554, 1.0982)	0.1229
FPG, mmol/L	0.0620	1.0640 (0.9971, 1.1350)	0.0613
TC, mmol/L	0.1001	1.1050 (1.0001, 1.2210)	0.0489
TG, mmol/L	0.0504	1.0520 (0.9703, 1.1400)	0.2200
HDL-c, mmol/L	0.1054	1.0540 (0.7486, 1.4840)	0.7630
LDL-c, mmol/L	0.0898	1.0940 (0.9714, 1.2320)	0.1390

[*] Average monthly income, [†] CNY: Chinese Yuan.

Results suggested that age, SBP, DBP, pulse pressure, WC, BMI, current smoking status, hypertension parental history, educational level and TC were related factors of hypertension ($P < 0.05$) in men. And the FPG was potentially related with hypertension in men (*P* value approximately 0.05).

Supplementary Table S4 Univariate Cox proportional hazards regression analysis for women in training set.

Variables	*β*	*HR (95%CI)*	*P*
Age, years	0.0388	1.0400 (1.0310, 1.0480)	<0.0001
SBP, mmHg	0.0847	1.0880 (1.0800, 1.0970)	<0.0001
DBP, mmHg	0.1077	1.1140 (1.1000, 1.1270)	<0.0001
Pulse pressure, mmHg	0.0642	1.0660 (1.0570, 1.0760)	<0.0001
WC, cm	0.0295	1.0300 (1.0220, 1.0390)	<0.0001
BMI, kg/m^2	0.0760	1.0790 (1.0540, 1.1040)	<0.0001
Heart rate, bpm*	0.0089	1.0090 (1.0010, 1.0170)	0.0286
T2DM (Yes vs No)	0.3966	1.4870 (1.1530, 1.9170)	0.0022
Drink (Yes vs No)	0.2189	1.2450 (0.4656, 3.3280)	0.6630
Higher vegetables and fruit intake (Yes vs No)	-0.1731	0.8411 (0.7109, 0.9950)	0.0435
High fat diet (Yes vs No)	-0.1200	0.8869 (0.4415, 1.7820)	0.7360
hypertension paternal history (Yes vs No)	0.1662	1.1810 (0.9969, 1.3390)	0.0543
General obesity (Yes vs No)	0.4136	1.5120 (1.2300, 1.8590)	<0.0001
Central obesity (Yes vs No)	0.4192	1.5210 (1.2910, 1.7920)	<0.0001
Weight categories			
Low weight	-0.0419	0.9589 (0.5088, 1.8070)	0.8970
Normal weight		Referent	
Over weight	0.4079	1.5037 (1.2573, 1.7980)	<0.0001
Obesity	0.6099	1.8402 (1.4636, 2.3140)	<0.0001
Income †, CNY ‡ (n, %)			
< 1000		Referent	
1000 ~	-0.3011	0.7400 (0.4834, 1.1330)	0.1660
≥ 3000	0.0165	1.0170 (0.5440, 1.9000)	0.9590
Educational level (n, %)			
Illiteracy		Referent	
Primary school	-0.4501	0.6376 (0.5195, 0.7825)	<0.0001
Junior high	-0.6825	0.5053 (0.4094, 0.6238)	<0.0001
High school and above	-0.5571	0.5729 (0.3989, 0.8228)	0.0026
FPG, mmol/L	0.0591	1.0610 (1.0160, 1.1080)	0.0075
TC, mmol/L	0.1349	1.1440 (1.0570, 1.2390)	0.0009
TG, mmol/L	0.1199	1.1274 (1.0570, 1.2020)	0.0002
HDL-c, mmol/L	-0.2421	0.7850 (0.6085, 1.0130)	0.0625
LDL-c, mmol/L	0.1222	1.1300 (1.0250, 1.2460)	0.0141

* bpm: beats per minute; † Average monthly income; ‡ CNY: Chinese Yuan.

Due to the fact that there are only nine current smokers in women (0.31 percent), the results of univariate analysis may lead to great random fluctuation, so current smoking is not considered in the women's univariate analysis.

In women, age, SBP, DBP, pulse pressure, WC, BMI, heart rates, fruit and vegetable intake, educational level, FPG, triglyceride (TG), low-density lipoprotein cholesterol (LDL-c) and TC were significantly related with hypertension ($P < 0.05$) based on the univariate Cox regression analysis. And the hypertension parental history and HDL-c were potentially related with hypertension in women (*P* value approximately 0.05).

Supplementary Table S5 Cross-validation of Cox regression models (M1, W1 and W2 model) for internal consistency of hypertension incident risk in training set.

Index	Index original	Training	Test	Optimism	Index corrected
**M1 model**					
**Dxy***	0.4773	0.4777	0.4711	0.0065	0.4707
**$R^{2\dagger}$**	0.1466	0.1473	0.1534	-0.0061	0.1527
**Slope**	1	1	0.9766	0.0234	0.9766
**D**	0.0453	0.0461	0.0631	-0.017	0.0623
**U**	-0.0003	-0.0004	0.0025	-0.0029	0.0026
**Q**	0.0456	0.0465	0.0606	-0.0141	0.0598
**g**	1.0541	1.0557	1.0295	0.0262	1.0279
**W1 model**					
**Dxy***	0.5622	0.5623	0.5587	0.0036	0.5586
**$R^{2\dagger}$**	0.1912	0.1918	0.2043	-0.0125	0.2037
**Slope**	1	1	1.0016	-0.0016	1.0016
**D**	0.0645	0.0655	0.0906	-0.0251	0.0896
**U**	-0.0002	-0.0002	0.0013	-0.0015	0.0013
**Q**	0.0647	0.0657	0.0893	-0.0235	0.0883
**g**	1.3769	1.3778	1.3772	0.0006	1.3763
**W2 model**					
**Dxy***	0.5612	0.5614	0.5564	0.005	0.5561
**$R^{2\dagger}$**	0.1926	0.1933	0.2058	-0.0125	0.2051
**Slope**	1	1	1.0088	-0.0088	1.0088
**D**	0.0651	0.0661	0.0925	-0.0264	0.0914
**U**	-0.0002	-0.0002	0.003	-0.0032	0.003
**Q**	0.0653	0.0663	0.0895	-0.0232	0.0885
**g**	1.3831	1.3848	1.3906	-0.0059	1.3889

***Dxy** means Somers' *Dxy*. When *Y* is binary, $Dxy = 2 \times (c - 0.5)$ where *c* is the concordance probability or area under the receiver operating characteristic curve, a linear translation of the Wilcoxon-Mann-Whitney statistic.

†$\boldsymbol{R^2}$ is mostly a measure of discrimination, and R^2_{adj} is a good overfitting-corrected measure, if the model is pre-specified. Estimating the relationship between the predicted probability and the observed outcome in calibration also leads to indexes of unreliability (U), discrimination (D), and overall quality (Q = D − U) which are derived from likelihood ratio tests. Q is a logarithmic scoring rule, which can be compared with Brier's index. The g-index is a new measure of a model's predictive discrimination, an interpretable, robust, and highly efficient measure of variation.

Supplementary Table S6 Discriminative ability, sensitivity and specificity of the different 6-year hypertension incident risk models for men in training set.

Models	AUC(95%*CI*)	Cut-off	Sensitivity(%)	Specificity(%)	Youden index
M1 [*†]	0.765 (0.745, 0.784)	0.1926	77.3	64.6	0.419
ANN [*†]	0.767 (0.747, 0.786)	0.2305	76.6	64.3	0.409
NBC [†‡§]	0.751 (0.730, 0.770)	0.2205	77.1	62.6	0.397
CART [*‡§]	0.720 (0.699, 0.741)	0.0994	79.6	56.1	0.357

[*] means AUC is statistically different compared with NBC model.

[†] means AUC is statistically different compared with CART model.

[‡] means AUC is statistically different compared with M1 model.

[§] means AUC is statistically different compared with ANN model.

CART model had a lower AUC compared with M1 model (P < 0.001), ANN model (P < 0.001) and NBC model (P = 0.001) for men. NBC model had a lower AUC compared with M1 model (P = 0.022) and ANN model (P = 0.019) for men.

Supplementary Table S7 Discriminative ability, sensitivity and specificity of the different 6-year hypertension incident risk models for women in training set.

Models	AUC(95%*CI*)	Cut-off	Sensitivity(%)	Specificity(%)	Youden index
W1 [*†]	0.806 (0.791, 0.820)	0.1920	74.3	73.7	0.480
W2 [*†]	0.806 (0.791, 0.820)	0.1922	74.0	74.2	0.482
ANN [*†]	0.809 (0.795, 0.823)	0.2512	74.0	75.8	0.498
NBC [†‡§‖]	0.796 (0.780, 0.810)	0.2588	74.8	72.7	0.475
CART [*‡§‖]	0.740 (0.724, 0.756)	0.0909	71.9	72.7	0.446

[*] means AUC is statistically different compared with NBC model.

[†] means AUC is statistically different compared with CART model.

[‡] means AUC is statistically different compared with W1 model.

[§] means AUC is statistically different compared with W2 model.

[‖] means AUC is statistically different compared with ANN model.

CART model had a lower AUC compared with W1 model, W2 model, ANN model and NBC model for women (P < 0.001). NBC model had a lower AUC compared with W1 model (P = 0.016), W2 mode (P = 0.019) and ANN model (P = 0.011) for women.

Supplementary Table S8 Calibration of Kaplan-Meier observed and predicted 6-year hypertension incident for men in training set.

Groups	Actual events*	Kaplan-Meier adjusted events†	Predicted events‡	Calibration χ^2	*P* value
**M1 model**					
1	7	8.133	6.314		
2	8	8.672	12.255		
3	17	18.223	17.177		
4	16	17.731	22.518		
5	23	25.704	28.929		
6	34	36.341	36.222	4.91334	0.84180
7	49	53.636	46.110		
8	56	59.259	57.212		
9	70	72.881	73.651		
10	95	98.253	98.331		
**ANN model**					
1	5	5.860	9.041		
2	9	9.920	15.833		
3	19	21.160	21.366		
4	15	15.950	27.595		
5	26	28.350	34.678		
6	31	34.290	43.003	24.54347	0.00352
7	46	49.270	52.938		
8	60	63.070	63.796		
9	59	61.650	77.499		
10	105	107.480	99.891		
**NBC model**					
1	7	8.083	1.218		
2	13	14.756	4.595		
3	17	18.314	9.667		
4	21	23.774	17.594		
5	16	17.397	29.081		
6	35	37.417	43.341	105.88180	<0.00001
7	48	51.223	62.927		
8	59	63.547	84.266		
9	62	65.009	107.362		
10	97	99.530	135.796		
**CART model**					
1	78	86.900	88.000		
2	130	138.300	154.000	4.56824	0.10186
3	167	174.300	190.000		

* Actual number of events through follow-up period.

† Observed number of events after Kaplan-Meier adjustment through follow-up period.

‡ Predicted number of events based on the different models through follow-up period.

Supplementary Table S9 Calibration of Kaplan-Meier observed and predicted 6-year hypertension incident for women in training set.

Groups	Actual events*	Kaplan-Meier adjusted events†	Predicted events‡	Calibration χ^2	*P* value
**W1 model**					
1	8	8.535	12.980		
2	38	41.079	36.910		
3	58	63.202	72.750	4.72712	0.31645
4	135	144.485	136.010		
5	249	261.600	257.850		
**W2 model**					
1	11	12.050	12.830		
2	33	35.440	36.600		
3	62	67.080	72.690	1.18206	0.88104
4	134	143.590	135.140		
5	248	260.950	259.130		
**ANN model**					
1	10	10.750	15.200		
2	30	32.420	39.620		
3	65	71.010	74.460	5.44370	0.24472
4	133	141.500	141.420		
5	250	263.350	245.540		
**NBC model**					
1	10	10.780	3.479		
2	34	37.240	22.294		
3	59	63.810	80.058	193.18980	<0.00001
4	147	155.890	211.037		
5	238	251.680	407.628		
**CART model**					
1	129	140.300	170.000		
2	199	209.300	240.000	17.95192	0.00012
3	160	169.600	194.000		

* Actual number of events through follow-up period.

† Observed number of events after Kaplan-Meier adjustment through follow-up period.

‡ Predicted number of events based on the different models through follow-up period.

Supplementary Table S10 Discriminative ability, sensitivity and specificity of the different 6-year hypertension incident risk models for men in testing set.

Models	AUC(95%*CI*)	Cut-off	Sensitivity(%)	Specificity(%)	Youden index
M1 *	0.771 (0.750, 0.791)	0.1745	83.3	59.7	0.430
ANN *	0.773 (0.752, 0.793)	0.2799	68.2	74.0	0.422
NBC *	0.760 (0.738, 0.781)	0.2205	78.2	62.5	0.407
CART †‡§	0.722 (0.699, 0.743)	0.0994	80.1	55.5	0.356

* means AUC is statistically different compared with CART model.

† means AUC is statistically different compared with M1 model.

‡ means AUC is statistically different compared with ANN model.

§ means AUC is statistically different compared with NBC model.

CART model had a lower AUC compared with M1 model, ANN model and NBC model for men ($P < 0.001$).

Supplementary Table S11 Discriminative ability, sensitivity and specificity of the different 6-year hypertension incident risk models for women in testing set.

Models	AUC(95%*CI*)	Cut-off	Sensitivity(%)	Specificity(%)	Youden index
W1 *	0.765 (0.746, 0.783)	0.1798	71.5	69.5	0.410
W2 *	0.764 (0.746, 0.783)	0.1446	79.6	61.6	0.412
ANN *	0.756 (0.737, 0.775)	0.2022	75.8	64.5	0.403
NBC *	0.761 (0.742, 0.779)	0.1860	78.3	63.1	0.414
CART †‡§‖	0.698 (0.677, 0.717)	0.0909	67.4	68.6	0.360

* means AUC is statistically different compared with CART model.

† means AUC is statistically different compared with W1 model.

‡ means AUC is statistically different compared with W2 model.

§ means AUC is statistically different compared with ANN model.

‖ means AUC is statistically different compared with NBC model.

CART model had a lower AUC compared with W1 model, W2 model, ANN model and NBC model for women ($P < 0.001$).

Supplementary Table S12 Calibration of Kaplan-Meier observed and predicted 6-year hypertension incident for men in testing set.

Groups	Actual events*	Kaplan-Meier adjusted events†	Predicted events‡	Calibration χ^2	***P*** **value**
**M1 model**					
1	6	7.145	5.465		
2	6	6.612	10.669		
3	12	12.800	14.919		
4	13	14.145	19.585		
5	22	24.732	25.246	6.30570	0.70898
6	30	32.536	31.939		
7	40	43.314	40.553		
8	53	55.694	50.087		
9	62	64.824	64.789		
10	80	82.800	85.824		
**ANN model**					
1	5	5.810	7.800		
2	7	7.885	13.810		
3	14	15.496	18.640		
4	11	11.615	24.190		
5	25	27.557	30.210	29.27430	0.00058
6	26	29.310	37.800		
7	40	42.909	46.440		
8	52	54.473	55.670		
9	51	53.556	67.990		
10	93	94.976	86.770		
**NBC model**					
1	5	6.031	1.021		
2	11	12.667	4.025		
3	11	11.923	8.625		
4	16	18.267	15.609		
5	18	19.974	25.613	82.26996	<0.00001
6	30	31.739	38.432		
7	40	42.337	55.350		
8	54	57.485	73.817		
9	54	56.913	94.303		
10	85	87.500	118.341		
**CART model**					
1	65	72.890	75.870		
2	112	118.570	134.950	5.249259	0.07247
3	147	154.000	168.830		

* Actual number of events through follow-up period.

† Observed number of events after Kaplan-Meier adjustment through follow-up period.

‡ Predicted number of events based on the different models through follow-up period.

Supplementary Table S13 Calibration of Kaplan-Meier observed and predicted 6-year hypertension incident for women in testing set.

Groups	Actual events*	Kaplan-Meier adjusted events†	Predicted events‡	Calibration χ^2	*P* value
**W1 model**					
1	13	14.02	10.78		
2	28	29.97	29.14		
3	60	66.56	55.69	6.78323	0.14780
4	109	115.22	100.41		
5	172	179.74	193.56		
**W2 model**					
1	13	13.790	10.630		
2	27	29.280	28.830		
3	65	71.020	55.690	7.40462	0.11599
4	104	110.420	100.370		
5	173	180.330	195.010		
**ANN model**					
1	15	15.950	12.520		
2	29	31.730	31.700		
3	66	72.070	57.660	4.74466	0.31451
4	103	110.170	103.690		
5	169	175.630	182.410		
**NBC model**					
1	17	18.450	3.347		
2	23	25.680	20.721		
3	64	67.970	64.790	189.75400	<0.00001
4	118	126.840	158.387		
5	160	166.540	299.485		
**CART model**					
1	121	129.900	115.000		
2	138	146.300	172.400	19.73303	0.00005
3	123	129.600	160.700		

* Actual number of events through follow-up period.

† Observed number of events after Kaplan-Meier adjustment through follow-up period.

‡ Predicted number of events based on the different models through follow-up period.

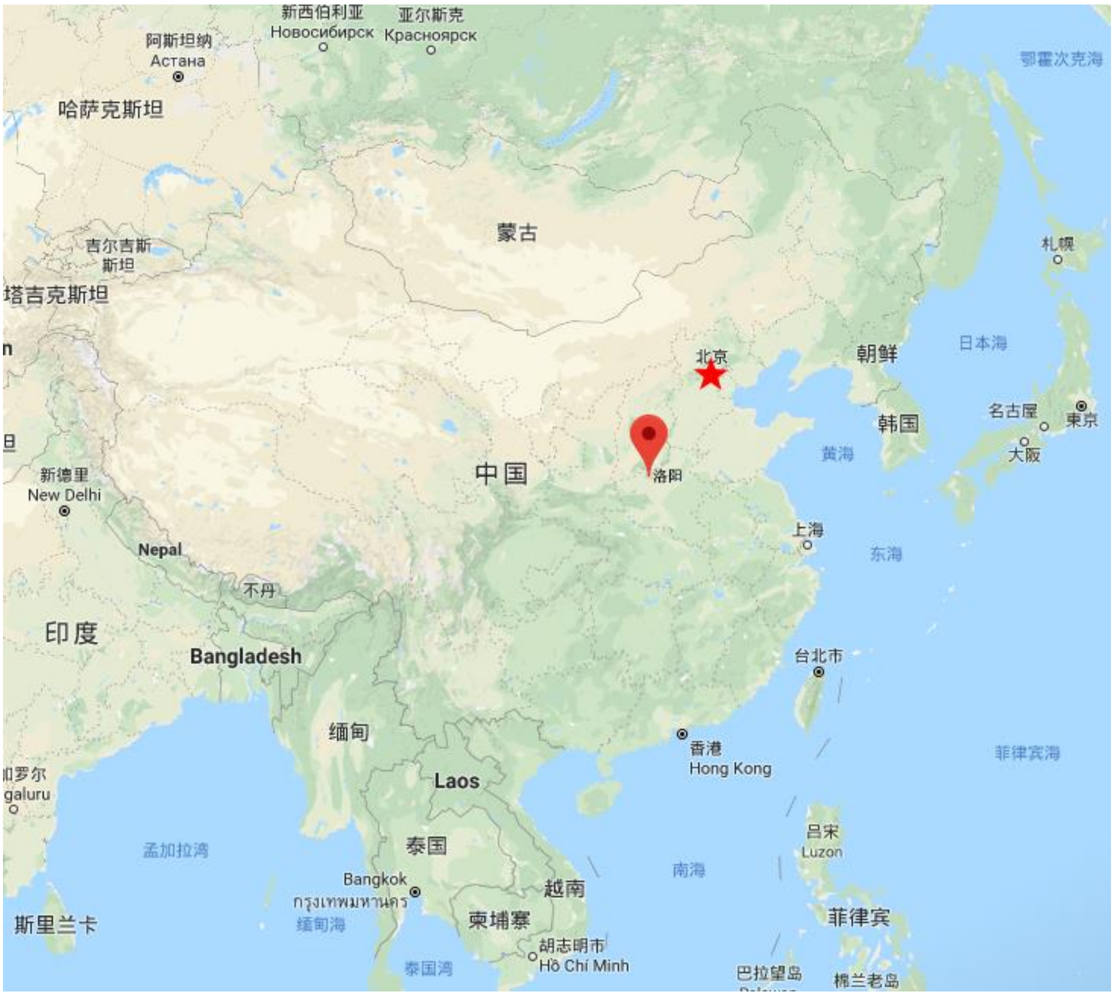

Supplementary Figure S1 Sketch map of data collection site. Red start shows the position of Beijing (capital of China) and the red location point shows the data collection site (Luoyang City).

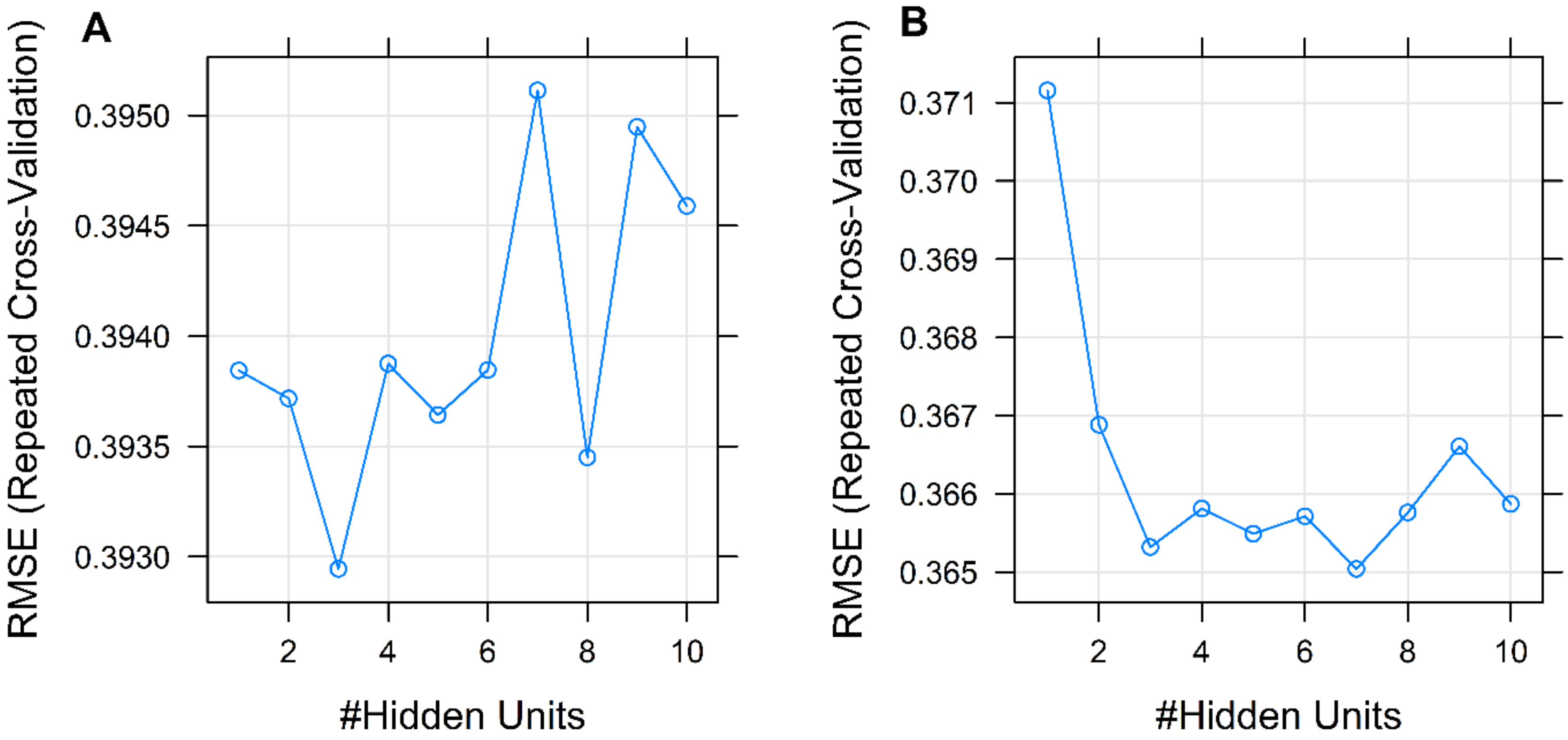

Supplementary Figure S2 Nodes in hidden layer for men (A) and women (B) in ANN models according to 10-fold cross-validation.

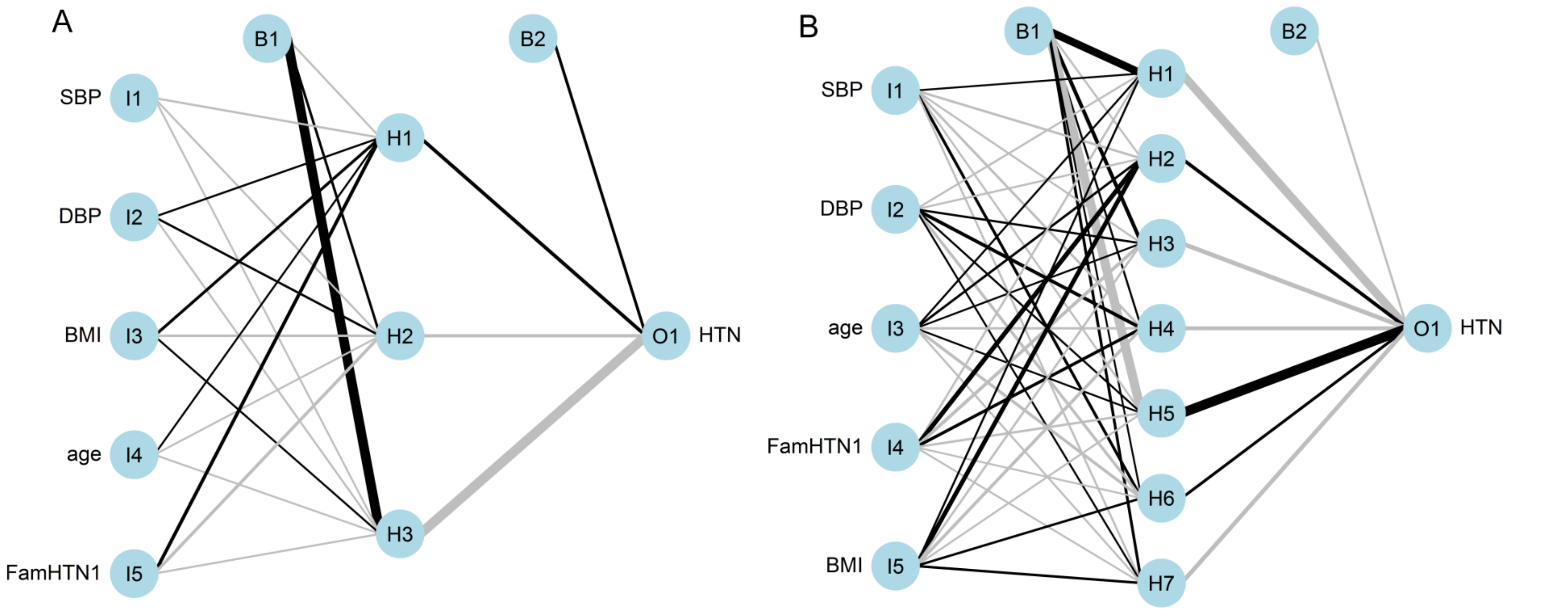

Supplementary Figure S3 Network chat of ANN models. There are three nodes in hidden layer for men (A) and nine nodes in hidden layer for women (B), and line thickness and color gradation represent the weight of the predictors. FamHTN1 means hypertension parental history and HTN means hypertension.

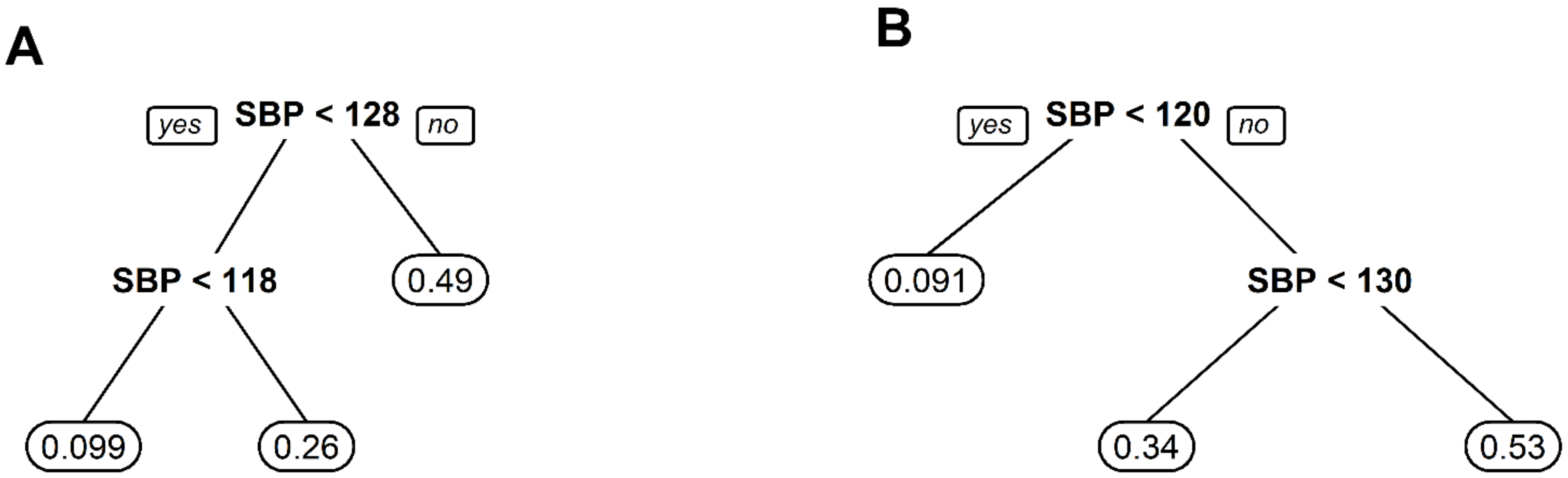

Supplementary Figure S4 Tree graph of CART models for men (A) and women (B).

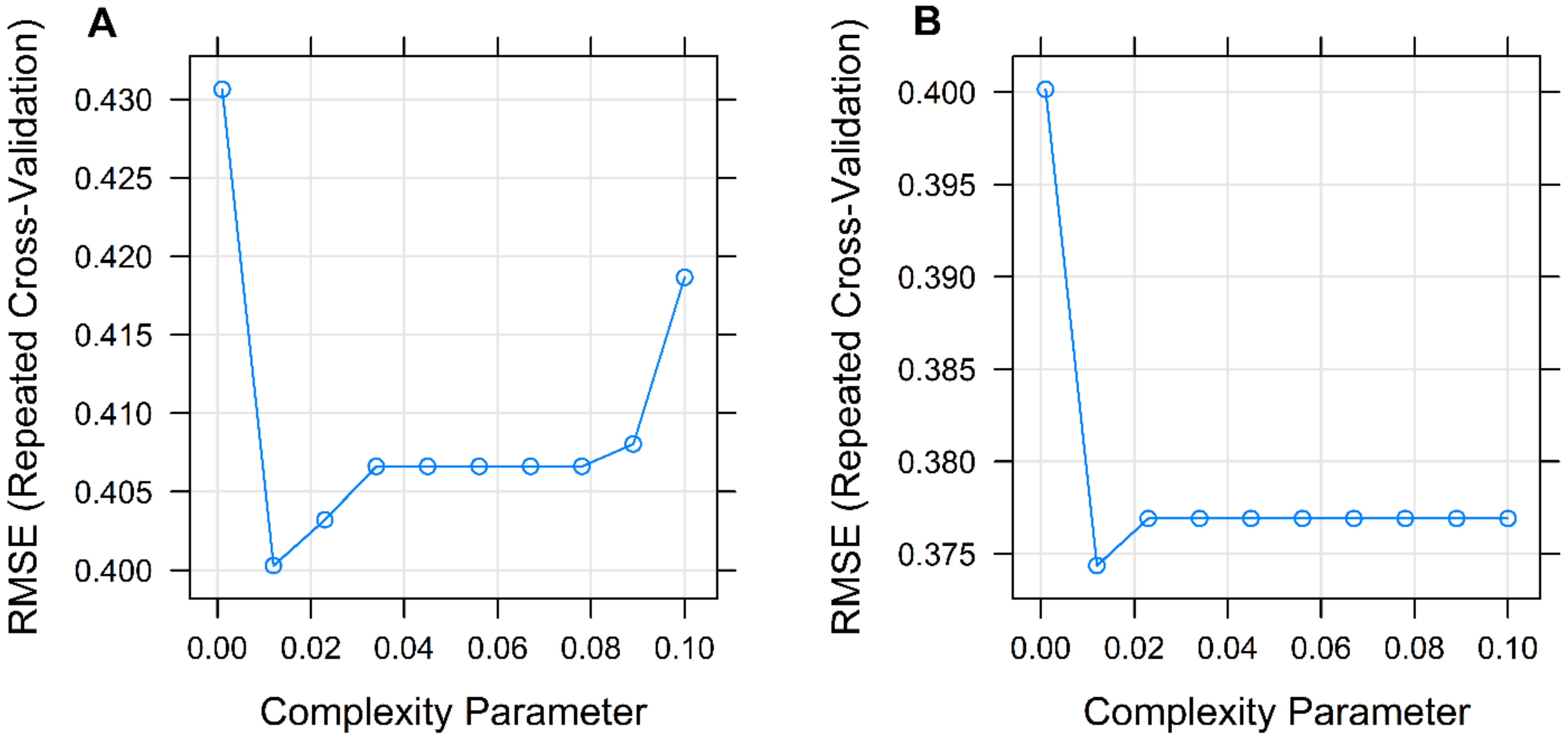

Supplementary Figure S5 Complexity parameter for men (A) and women (B) in CART models according to 10-fold cross-validation.

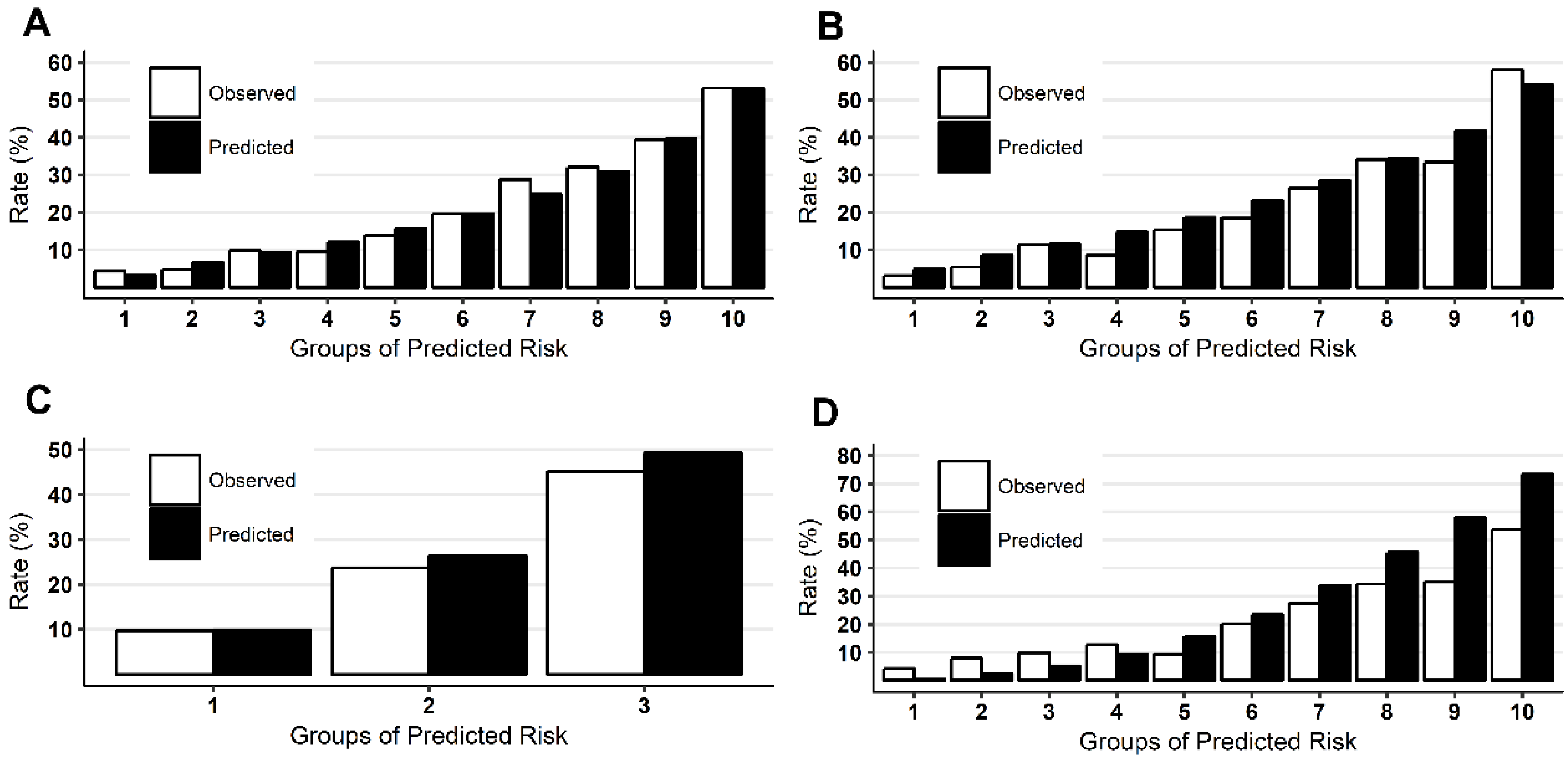

Supplementary Figure S6 Kaplan-Meier observed and predicted hypertension incident for different hypertension models in training set men: M1 model (A), ANN model (B), CART model (C) and NBC model (D), by groups of predicted probabilities.

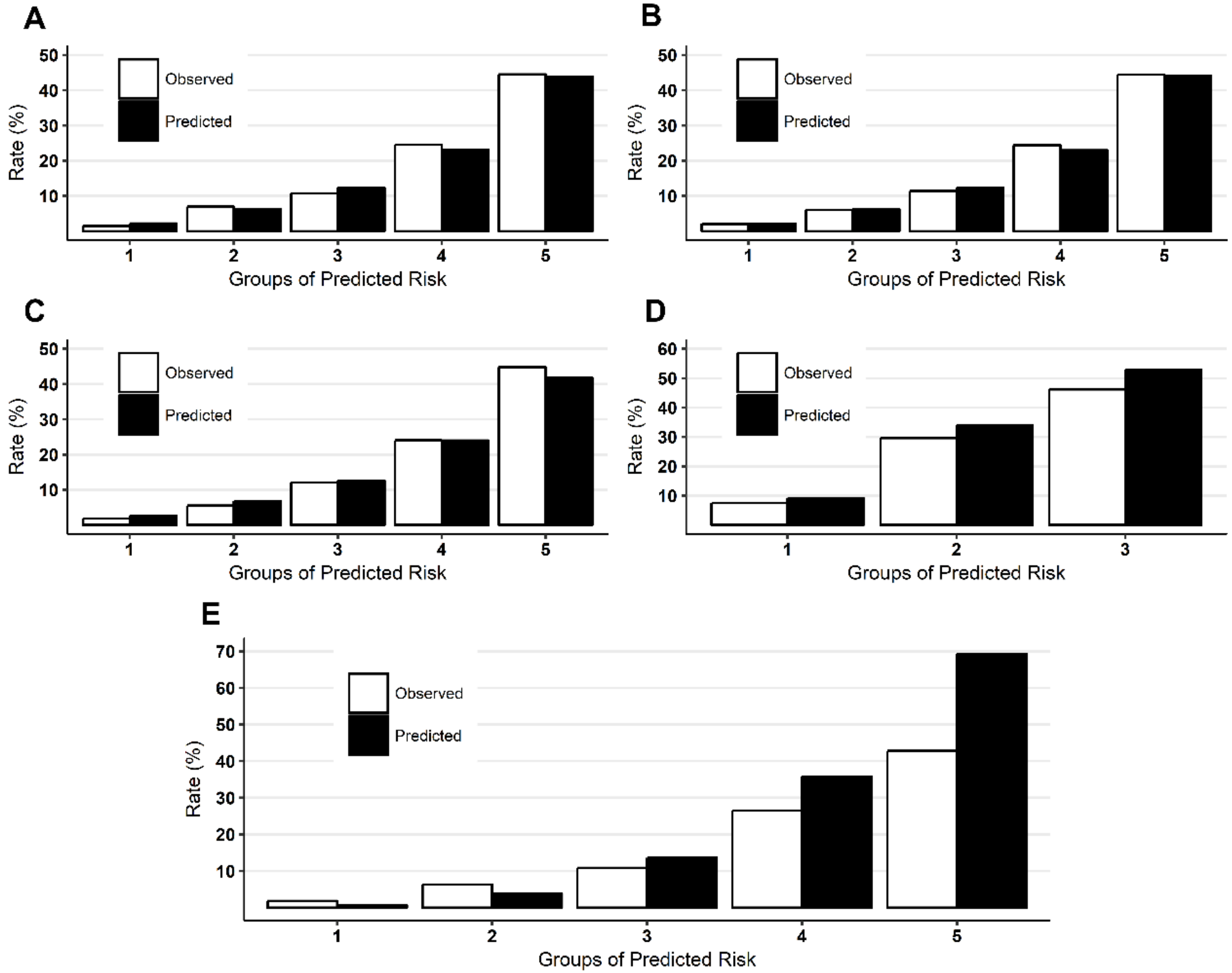

Supplementary Figure S7 Kaplan-Meier observed and predicted hypertension incident for different hypertension models in training set women: W1 model (A), W2 model (B), ANN model (C), CART model (D) and NBC model (E), by groups of predicted probabilities.

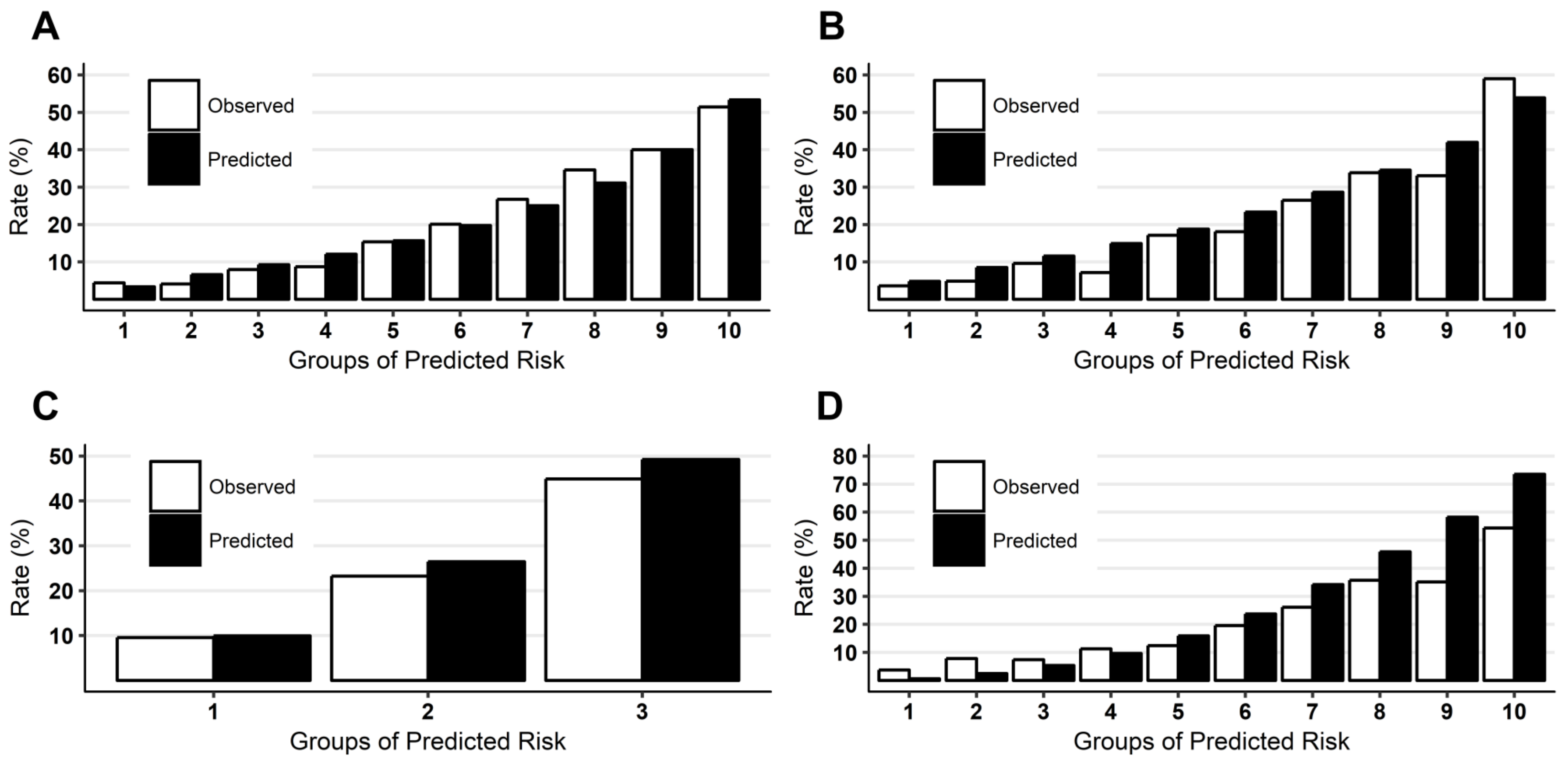

Supplementary Figure S8 Kaplan-Meier observed and predicted hypertension incident for different hypertension models in testing set men: M1 model (A), ANN model (B), CART model (C) and NBC model (D), by groups of predicted probabilities.

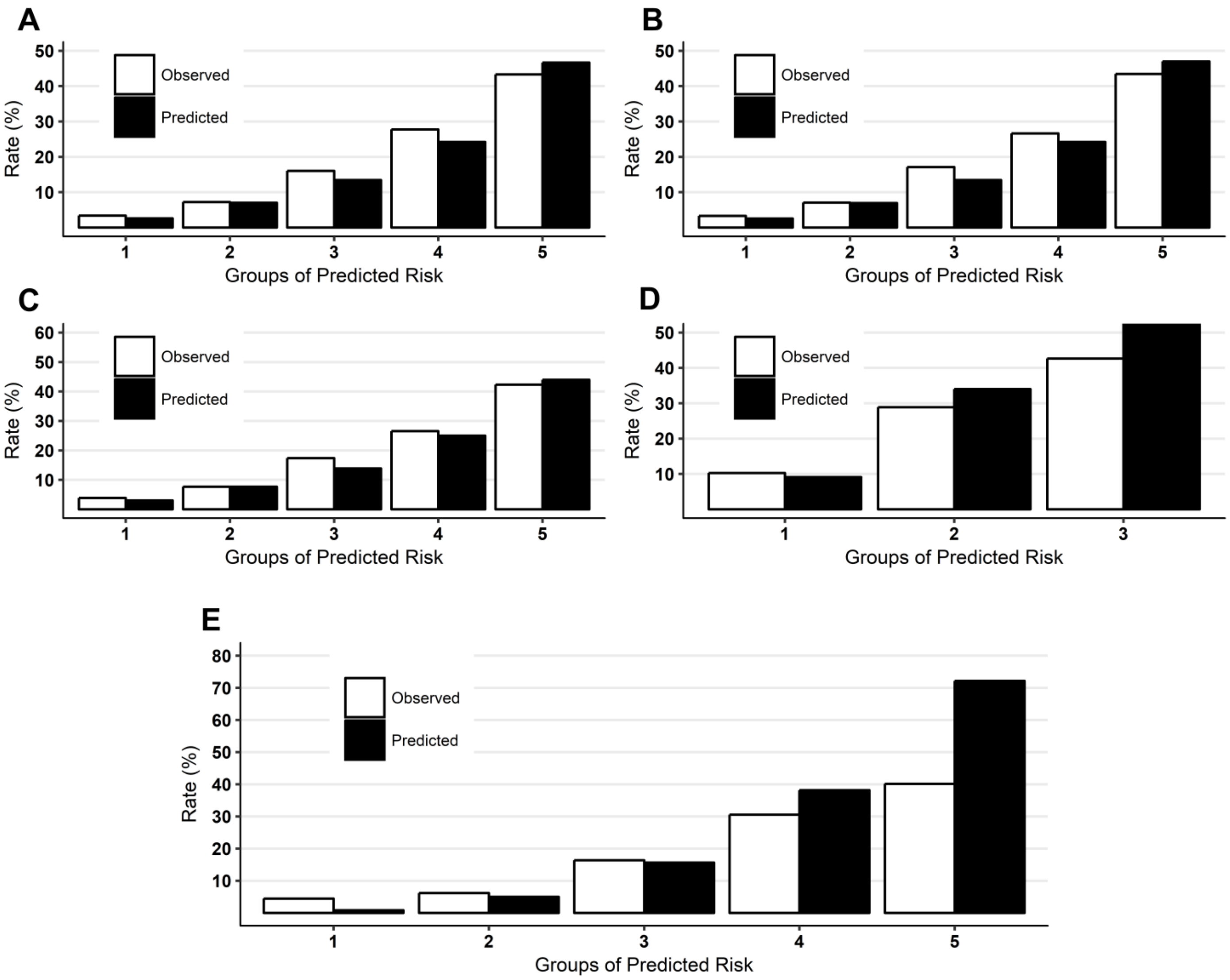

Supplementary Figure S9 Kaplan-Meier observed and predicted hypertension incident for different hypertension models in testing set women: W1 model (A), W2 model (B), ANN model (C), CART model (D) and NBC model (E), by groups of predicted probabilities.
